# Risk factors of atrial fibrillation occurring after radical surgery of esophageal carcinoma

**DOI:** 10.1186/s13019-019-0885-z

**Published:** 2019-03-14

**Authors:** Jun Tang, Jian-zhu Zhao, Kai-ming Ren, Fu-shuang Zheng, Xi-wen Wang, Hai-jun Liu, Jun-gang Zhao, Ji-bin Lu

**Affiliations:** 1 Department of Thoracic Surgery, Shengjing Hospital of China Medical University, Shenyang, 110004 China; 2 Department of Oncology, Shengjing Hospital of China Medical University, No 36, Sanhao Street, Herping District, Shenyang, 110004 China

**Keywords:** Radical surgery of esophageal cancer, Atrial fibrillation, Risk factors

## Abstract

**Background:**

Atrial fibrillation (AF) is a common complication after radical surgery of esophageal cancer. The aim of this study was to explore AF risk factors after radical surgery of esophageal carcinoma.

**Method:**

The data of 335 patients with esophageal cancer who were admitted in our hospital from January 2014 to August 2016 for the first time were retrospectively analyzed. We retrieved the papers in some data banks using the search terms including English and Chinese search terms, and obtained 13 factors which were mentioned in more than 6 papers. The 13 factors including age, gender, history of smoking, history of hypertension, history of peripheral vascular disease, history of cardiac stents or angina pectoris, preoperative pulmonary infection, preoperative brain natriuretic peptide (BNP) level, preoperative left ventricular diastolic dysfunction, operative method, lesion location, intraoperative blood transfusion, adhesion between lymph nodes and pericardium, underwent univariate and multivariate analyses.

**Results:**

Of the 335 patients with esophageal cancer, 48 had AF within one week after operation. Univariate analysis indicated that the age (*OR*: 4.89; *CI*: 2.53–9.47, *P*: 0.000), gender (*OR*: 2.26; *CI*: 1.17–4.37, *P*: 0.013), history of peripheral vascular disease (*OR*: 2.29; *CI*: 1.06–4.92, *P*: 0.030), history of cardiac stents or angina pectoris (*OR*: 27.30; *CI*: 12.44–59.91, *P*: 0.000), preoperative BNP level (*OR*: 27.13; *CI*: 10.97–67.06, *P*: 0.000), preoperative left ventricular diastolic dysfunction (*OR*: 2.22; *CI*: 1.19–4.14, *P*: 0.012), operative method (*OR*: 2.09; *CI*: 1.002–4.380, *P*: 0.046), intraoperative blood transfusion (*OR*: 20.24; *CI*: 8.39–48.82, *P*: 0.000), and adhesion between lymph nodes and pericardium were risk factors (*OR*: 2.05; *CI*: 1.08–3.87, *P*: 0.024). Furthermore, multivariate analysis displayed that advanced age (*OR*: 5.044; *CI*: 1.748–14.554, *P*: 0.003), male (*OR*: 6.161; *CI*: 2.143–17.715, *P*: 0.001), history of cardiac stents or angina pectoris (*OR*: 48.813; *CI*: 13.674–174.246, *P*: 0.000), preoperative BNP > 100 (*OR*: 41.515; *CI*: 9.380–183.732, *P*: 0.000), open surgery (*OR*: 3.357; *CI*: 1.026–10.983, *P*: 0.045), intraoperative blood transfusion (*OR*: 58.404; *CI*: 10.777–316.509, *P*: 0.000), and adhesion between lymph nodes and pericardium (*OR*: 3.954; *CI*: 1.364–11.459, *P*: 0.011) were risk factors which could increase the incidence of postoperative AF.

**Conclusion:**

We should pay attention to the above risk factors in order to reduce the incidence of postoperative AF.

## Introduction

Postoperative atrial fibrillation (AF) has been one of the most common complications after general thoracic surgery. As early as 1974, M. Haimov, and S. Glabman reported a high incidence of AF after general thoracic surgery [1]. Most studies have shown that the incidence of postoperative AF is between 4 and 37% [2]. It usually occurs in an early stage (1–3 days) after radical surgery of esophageal cancer [3]. Postoperative AF will further increase the risk of stroke or thrombosis, mortality, hospital day and health care cost [4]. Paying attention to these high risk factors can decrease both the incidence of postoperative AF and the cost of hospitalization. In this study, many potential risk factors related to AF occurring after radical surgery of esophageal cancer were analyzed to find out the risk factors of postoperative AF, providing a basis for prevention of postoperative AF in clinical practice.

## Materials and methods

### Subjects

A total of 375 consecutive patients who underwent radical surgery of esophageal cancer in our hospital between January 2014 and August 2016 for the first time were collected. The inclusion criteria were (1) age >18 years and (2) the patients receiving radical surgery of esophageal cancer. The exclusion criteria were (1) no rigorous preoperative evaluation (7 patients); (2) a history of cardiac arrhythmias before surgery (21 patients) and (3) no electrocardiogram monitoring within 1 week after operation (12 patients). The clinical data of 335 patients in line with the above inclusion and exclusion criteria were included in this retrospective study. Of the 335 patients, 262 were male and 73 female, with a mean of 63 years (range 40–88). In the 335 patients, 122 were over 70 years old; 262 were male, 202 had a history of smoking, 109 had a history of hypertension, 44 had a history of peripheral vascular disease, 42 had a history of cardiac stents or angina pectoris, 41 had preoperative pulmonary infection, 38 had preoperative BNP ≥100, 107 had preoperative left ventricular diastolic dysfunction, 223 received open surgery, 182 had esophageal cancer in the upper middle segment, 28 received intraoperative blood transfusion and 166 had adhesion between lymph nodes and pericardium.

### Diagnostic criteria of postoperative AF

The postoperative AF was diagnosed according to the following 3 items: (1) The AF occurred within 1 week after radical surgery of esophageal cancer; (2) The AF was confirmed by electrocardiogram; and (3) The AF required drug intervention.

### Factors related to postoperative AF

We retrieved the papers in the data banks including PubMed (January, 1992 to August, 2016), OVID Evidence-Based Medicine Database (January, 1994 to August, 2016), full-text database of Chinese journals (January, 1994 to August, 2016), VIP database (January, 1994 to August, 2016) and Wanfang database (January 1994 to August, 2016) using the search terms including English search terms such as esophageal cancer, atrial fibrillation, radical surgery and risk factors, as well as Chinese search terms such as食管癌, 房颤, 根治手术 and风险因素. We obtained 78 papers about AF risk factors after radical surgery of esophageal cancer. There were 13 factors which were mentioned in more than 6 papers, so the 13 factors were used as suspicious risk factors of postoperative AF. The 13 factors included age, gender, history of smoking, history of hypertension, history of peripheral vascular disease, history of cardiac stents or angina pectoris, preoperative pulmonary infection, preoperative brain natriuretic peptide (BNP) level, preoperative left ventricular diastolic dysfunction, operative method, lesion location, intraoperative blood transfusion and adhesion between lymph nodes and pericardium.

### Treatment of postoperative AF

The patients who had postoperative AF were treated. In details, for the patients who had AF within 24 h after radical surgery of esophageal cancer, cordarone was directly used for cardioversion of AF to sinus rhythm; for the patients who had AF 24 h after radical surgery of esophageal cancer, cordarone was used for cardioversion of AF to sinus rhythm after atrial thrombosis was excluded or patients received 3-week anticoagulant; and for the patients who had recurrent attacks of AF for 3 times or over; radiofrequency ablation was used for cardioversion of AF to sinus rhythm.

### Statistical analysis

SPSS 18.0 statistical software was used for statistical analysis. All the 13 suspicious risk factors were analyzed by univariate analysis. The factors which showed statistically significant differences in the univariate analysis were further analyzed by multivariate logistic regression analysis. The difference was statistically significant at *P* < 0.05.

## Results

### Clinical outcomes

AF occurred within 24 h after radical surgery of esophageal cancer in 15 cases, within 2 to 3 days after radical surgery of esophageal cancer in 19 cases, within 4 to 5 days after radical surgery of esophageal cancer in 8 cases and 6 to 7 days after radical surgery of esophageal cancer in 6 cases.

Of the 48 patients with postoperative AF, successful cardioversion of AF to sinus rhythm was performed by cordarone on 47 patients and by radiofrequency ablation on one patient.

### Univariate analysis

The univariate analysis indicated that there were significant differences in the proportion of postoperative AF between over 70-year-old patients (27%) and under 70-year-old patients (7%) (*OR*: 4.89; *CI*: 2.53–9.47, *P*: 0.000), male patients (23.3%) and female patients (11.0%) (*OR*: 2.26; *CI*: 1.17–4.37, *P*: 0.013), the patients with a history of peripheral vascular disease (25.0%) and the patients without a history of peripheral vascular disease (12.7%) (*OR*: 2.29; *CI*: 1.06–4.92, *P*: 0.030), the patients with a history of cardiac stents or angina pectoris (66.7%) and the patients without a history of cardiac stents or angina pectoris (6.8%) (*OR*: 27.30; *CI*: 12.44–59.91, *P*: 0.000), the patients with preoperative BNP ≥100 (72.4%) and the patients with preoperative BNP < 100 (8.8%) (*OR*: 27.13; *CI*: 10.97–67.06, *P*: 0.000), the patients with preoperative left ventricular diastolic dysfunction (21.5%) and the patients without preoperative left ventricular diastolic dysfunction (11.0%) (*OR*: 2.22; *CI*: 1.19–4.14, *P*: 0.012), the patients receiving open surgery (17.0%) and the patients receiving thoracoscopic surgery (8.9%) (*OR*: 2.09; *CI*: 1.002–4.380, *P*: 0.046), the patients receiving intraoperative blood transfusion (67.9%) and the patients not requiring intraoperative blood transfusion (9.4%) (*OR*: 20.24; *CI*: 8.39–48.82, *P*: 0.000), as well as the patients with adhesion between lymph nodes and pericardium (18.7%) and the patients without adhesion between lymph nodes and pericardium (10.1%) (*OR*: 2.05; *CI*: 1.08–3.87, *P*: 0.024).

By contrast, the univariate analysis also displayed that there were no significant differences in the proportion of postoperative AF between the patients with a history of smoking (14.4%) and the patients without a history of smoking (14.3%) (*OR*: 1.006; *CI*: 0.538–1.880, *P*: 0.986), the patients with a history of hypertension (18.3%) and the patients without a history of hypertension (12.4%) (*OR*: 1.59; *CI*: 0.85–2.97, *P*: 0.145), the patients with preoperative pulmonary infection (14.6%) the patients without preoperative pulmonary infection (14.3%) (*OR*: 1.03; *CI*: 0.41–2.59, *P*: 0.952), as well as the patients with esophageal cancer occurring in the upper middle segment (13.7%) and the patients with esophageal cancer occurring in the lower segment (15.0%) (*OR*: 0.900; *CI*: 0.488–1.660, *P*: 0.736) (Table 1).

**Table 1:** Results of univariate analysis [n (%)]

Factors	No AF(*n* = 287)	AF(*n* = 48)	χ^2^	*P* values	*OR*	95% *CI*
Age						
< 70	198 (93%)	15 (7%)	25.29	0.000	4.89	(2.53,9.47)
≥ 70	89 (73%)	33 (27%)				
Gender						
Male	201 (76.7%)	61 (23.3%)	6.104	0.013	2.26	(1.17,4.37)
Female	65 (89.0%)	8 (11.0%)				
History of smoking						
No	114 (85.7%)	19 (14.3%)	0.000	0.986	1.006	(0.538,1.880)
Yes	173 (85.6%)	29 (14.4%)				
Hypertension						
No	198 (87.6%)	28 (12.4%)	2.127	0.145	1.59	(0.85,2.97)
Yes	89 (81.7%)	20 (18.3%)				
History of peripheral vascular disease						
No	254 (87.3%)	37 (12.7%)	4.699	0.030	2.29	(1.06,4.92)
Yes	33 (75%)	11 (25.0%)				
History of cardiac stents or angina pectoris						
No	273 (93.2%)	20 (6.8%)	107.16	0.000	27.30	(12.44,59.91)
Yes	14 (33.3%)	28 (66.7%)				
Preoperative pulmonary infection						
No	252 (85.7%)	42 (14.3%)	0.004	0.952	1.03	(0.41,2.59)
Yes	35 (85.4%)	6 (14.6%)				
Preoperative BNP						
< 100	279 (91.2%)	27 (8.8%)	87.261	0.000	27.13	(10.97,67.06)
≥ 100	8 (27.6%)	21 (72.4%)				
Preoperative left-ventricular diastolic dysfunction						
No	203 (89.0%)	25 (11.0%)	6.579	0.012	2.22	(1.19,4.14)
Yes	84 (78.5%)	23 (21.5%)				
Operative methods						
Combined	102 (91.1%)	10 (8.9%)	3.996	0.046	2.09	(1.002,4.380)
Open	185 (83.0%)	38 (17.0%)				
Lesion location						
Lower	130 (85%)	23 (15.0%)	0.114	0.736	0.900	(0.488,1.660)
Middle or upper	157 (86.3%)	25 (13.7%)				
Blood transfusion						
No	278 (90.6%)	29 (9.4%)	71.31	0.000	20.24	(8.39,48.82)
Yes	9 (32.1%)	19 (67.9%)				
Adhesion to pericardium						
No	152 (89.9%)	17 (10.1%)	5.064	0.024	2.05	(1.08,3.87)
Yes	135 (81.3%)	31 (18.7%)				

Notes: *AF* Atrial fibrillation, *BNP* Brain natriuretic peptide, *OR* odds ratio, *CI* confidence interval

### Multivariate analysis

After univariate analysis, the factors with values *P* > 0.05 were excluded. The remaining factors with values *P* < 0.05 were put into the logistic regression equation, proving that this regression equation was meaningful. Subsequently, multivariate analysis showed that age (*OR*: 5.044; *CI*: 1.748–14.554, *P*: 0.003), gender (*OR*: 6.161; *CI*: 2.143–17.715, *P*: 0.001), history of cardiac stents or angina pectoris (*OR*: 48.813; *CI*: 13.674–174.246, *P*: 0.000), preoperative BNP (*OR*: 41.515; *CI*: 9.380–183.732, *P*: 0.000), surgical methods (*OR*: 3.357; *CI*: 1.026–10.983, *P*: 0.045), intraoperative blood transfusion (*OR*: 58.404; *CI*: 10.777–316.509, *P*: 0.000), as well as adhesion between lymph nodes and pericardium (*OR*: 3.954; *CI*: 1.364–11.459, *P*: 0.011) were the risk factors of postoperative AF (Table 2).

**Table 2:** Result of multivariate logistic regression analysis

Factors	B	S.E	Wald	*P*	*OR*	95% *CI*
Age	1.618	0.541	8.957	0.003	5.044	(1.748,14.554)
Gender	1.818	0.539	11.384	0.001	6.161	(2.143,17.715)
History of peripheral vascular disease	0.584	0.830	0.495	0.482	1.793	(0.352,9.124)
History of cardiac stents or angina pectoris	3.888	0.649	35.864	0.000	48.813	(13.674,174.246)
Preoperative BNP	3.726	0.759	24.106	0.000	41.515	(9.380,183.732)
Preoperative left-ventricular diastolic dysfunction	0.926	0.604	2.349	0.125	2.524	(0.773,8.244)
Operative methods	1.211	0.605	4.010	0.045	3.357	(1.026,10.983)
Blood transfusion	4.067	0.862	22.252	0.000	58.404	(10.777,316.509)
Adhesion to pericardiaum	1.375	0.543	6.409	0.011	3.954	(1.364,11.459)

Note: *BNP* Brain natriuretic peptide, *OR* odds ratio, *CI* confidence interval

The distributions of these risk factors in the patients are shown in Table 3.

**Table 3:** Distributions of risk factors of postoperative atrial fibrillation in these patients

	Blood transfusion	History of cardiac stents or angina pectoris	Preoperative BNP ≥ 100	Male	Over 70-year-old age	Adhesion to pericardium	Open surgery
Patients (n)	28	42	29	262	122	166	223

Note: *BNP* Brain natriuretic peptide

These factors increasing the risk of postoperative AF were as follows in the order from high to low: the intraoperative blood transfusion was 58.404 times, the history of cardiac stents or angina pectoris was 48.813 times, the preoperative BNP ≥ 100 was 41.515 times, the male was 6.161 times, the over 70-year-old age was 5.044 times, the adhesion between lymph nodes and pericardium was 3.954 times and the open surgery was 3.357 times (Table 2).

## Discussion

Although clinicians have tried to reduce the occurrence of postoperative AF, it is still the most common complication after radical surgery of esophageal cancer. In this study, 14.3% of patients were complicated with postoperative AF. Nearly one-third (15 cases) of these patients developed atrial fibrillation within the first 24 h after surgery, 70.8% (34 cases) occurred within 3 days after surgery, and only 12.5% (6 cases) occurred 5 days after surgery. These data suggest that dynamic postoperative electrocardiogram is necessary, especially for the patients with risk factors of postoperative AF.

The results of this study showed that the risk of postoperative AF in over 70-year-old patients was 5.044 times the risk of postoperative AF in under 70-year-old patients. For aged people, their atrial and ventricular structures, systolic and diastolic functions, as well as electrocardio conduction have undergone structural changes. An epidemiological survey says that AF prevalence rates were more than 1%, more than 5 and 10% in the people aged over 60 years, over 70 years and 80 years, respectively [5]. Surgery itself is an injury to the body and advanced age has always been a leading risk factor for perioperative cardiac complications. Amar et al. [6] observed the clinical data of 527 patients undergoing general thoracic surgery, and found that the incidence of postoperative AF was 4, 8, 14 and 25% in the patients aged under 50, 50–60, 60–70 and over 70 years, respectively. Their results are similar to that in this study and confirm that the incidence of postoperative AF increases with age.

Vaporciyan et al. [7] studied 2588 patients undergoing noncardiac thoracic surgery and believed that the risk factor of male sex may be related to the mechanism of immune response. Angele et al. [8] have proposed a hypothesis that the pro-inflammatory immune response caused by tissue trauma is more severe in male individuals than in female individuals, which is likely to lead to a higher incidence of postoperative AF in men than in women after surgical trauma.

The results of this study showed that a history of cardiac stents or angina pectoris was a risk factor for the occurrence of AF after radical surgery of esophageal cancer, and the risk of postoperative AF in the patients with a history of heart stents or angina pectoris was 48.813 times the risk of postoperative AF in the patients without a history of heart stents or angina pectoris. There has also been a report of increased risk of postoperative cardiac complications in patients with underlying heart disease [9], which may be caused by the enlargement of atrial chamber, the pathological changes in the cardiac pacemaker conduction, and even the poor ability of cardiac function compensation and intolerance to anesthesia and surgery [10]. Therefore, there is no doubt that these patients are at high risk of perioperative arrhythmias, and a significant increase in the incidence of postoperative AF is understandable. These patients need to stop using anticoagulants more than 1 week before operation and preventive use of antiarrhythmic drugs is of certain significance [11].

Toufektzian et al. [12] reported that preoperative BNP was associated with AF after general thoracic surgery. The results of this study showed that the risk of postoperative AF in the patients with preoperative BNP ≥100 was 41.515 times the risk of postoperative AF in the patients with preoperative BNP < 100. The patients with high BNP may not show the symptoms of heart failure before operation, but they may have got subclinical manifestations such as left atrial dilatation, cardiac dysfunction and so on [13]. N-terminal brain natriuretic peptide precursor (NT pro-BNP) is more susceptible to age, sex, and kidney function [14]. More and more data have proved that the increase of pre-operative BNP indicates the occurrence of postoperative cardiovascular adverse events, especially AF [15, 16]. Therefore, BNP was a possible risk factor in this study. Generally speaking, BNP elevation indicates heart failure or atrial and ventricular wall dilatation, but it is also affected by age, atrial fibrosis, inflammation and unknown factors.

The results of this study indicated that the risk of postoperative AF in the patients receiving open surgery was 3.357 times the risk of postoperative AF in the patients receiving combined thoracoscopic surgery, which may be related to great trauma, heavy pain, slow recovery and other complications of open surgery. Compared with the patients receiving minimally invasive esophageal cancer surgery, the incidence of AF was increased in the patients receiving open esophageal cancer surgery [17]. The results of this study also showed that the risk of postoperative AF in the patients with intraoperative blood transfusion were 58.404 times the risk of postoperative AF in the patients without intraoperative blood transfusion and intraoperative blood transfusion was the biggest risk factor obtained in this study. Generally speaking, all operations requiring blood transfusion are complicated with bleeding, trauma, collateral damage and postoperative complications. Blood transfusion is easy to be complicated with AF after surgery due to insufficient fluid volume, electrolyte disturbance, immune changes and other factors [18]. Moskowitz et al. [19] suggests that debleaching red blood cells in mice may relieve severe inflammatory reactions and decrease the expression levels of various proinflammatory cytokines, which is the evidence that blood transfusions may lead to elevation of inflammation level.

It has been reported in many literatures that pericardial operation may increase the risk of AF [20–22]. In order to reveal the operative field, routine lymph node resection (subcarinal lymph nodes, adjacent esophageal lymph node and diaphragmatic lymph nodes) usually squeezes the pericardium, which is likely to lead to cardiac injury with the occurrence of postoperative AF. This study suggests that the risk of postoperative AF in the patients with adhesion between lymph nodes and pericardium was 3.954 times the risk of postoperative AF in the patients without adhesion, which seems to be consistent with the reported results above to some extent.

In this study, it is worth paying attention to that hypertension is not a risk factor of postoperative AF, which is different from other reports. Hypertension is a primary risk factor of AF [23], and approximately 53% of patients with AF have hypertension [24]. While, in this study, 32.5% of the patients had a history of hypertension before operation, and univariate analysis indicated that there was no statistically significant difference in the incidence rate of postoperative AF between the patients with and without a history of hypertension. This may be related to the insufficient sample size of this study.

The advantages of this study are as follows: Firstly, we observed only a complication (AF), and its risk factors were after receiving the same type of surgery (radical resection of esophageal cancer) in the same disease (esophageal cancer). Secondly, the dynamic review of electrocardiogram in this study strongly confirmed the diagnosis of AF. Finally, the risk factors in this study are all quantifiable factors, which are easy to attract the attention of surgeons and anesthesiologists before operation.

There are some limitations in this study as follows: Firstly, this retrospective study based on a single institution had its own limitations in controlling bias and confounding factors, as well as establishing causality and universality. Secondly, this study did not collect possible risk factors of postoperative AF such as electrolytes, hypoxia, anemia, hypothermia and other clinical data for statistical analysis, which may cause the interference of the results. Thirdly, because this study is a clinical study, it is not possible to deeply explore the molecular biological mechanism of these risk factors.

## Conclusions

In summary, advanced age, male, a history of cardiac stents or angina pectoris, preoperative BNP ≥100, open surgery, intraoperative blood transfusion and the adhesion between lymph nodes and pericardium are the risk factors associated with AF after radical surgery of esophageal carcinoma. Effective preoperative prevention for the above factors is likely to reduce the incidence of postoperative AF and greatly improve the prognosis of patients.

## Abbreviations

AF: Atrial fibrillation
BNP: Brain natriuretic peptide
